# Manual Versus Artificial Intelligence-Based Segmentations as a Pre-processing Step in Whole-body PET Dosimetry Calculations

**DOI:** 10.1007/s11307-022-01775-5

**Published:** 2022-10-04

**Authors:** Joyce van Sluis, Walter Noordzij, Elisabeth G. E. de Vries, Iris C. Kok, Derk Jan A. de Groot, Mathilde Jalving, Marjolijn N. Lub-de Hooge, Adrienne H. Brouwers, Ronald Boellaard

**Affiliations:** 1 Department of Nuclear Medicine and Molecular Imaging, University Medical Center Groningen, University of Groningen, Groningen, The Netherlands; 2 Department of Medical Oncology, University Medical Center Groningen, University of Groningen, Groningen, The Netherlands; 3 Department of Clinical Pharmacy and Pharmacology, University Medical Center Groningen, University of Groningen, Groningen, The Netherlands; 4 Department of Radiology and Nuclear Medicine, Cancer Center Amsterdam, Amsterdam UMC, Vrije Universiteit Amsterdam, Amsterdam, The Netherlands

**Keywords:** Dosimetry, Segmentation, Artificial intelligence

## Abstract

**Purpose:**

As novel tracers are continuously under development, it is important to obtain reliable radiation dose estimates to optimize the amount of activity that can be administered while keeping radiation burden within acceptable limits.

Organ segmentation is required for quantification of specific uptake in organs of interest and whole-body dosimetry but is a time-consuming task which induces high interobserver variability. Therefore, we explored using manual segmentations versus an artificial intelligence (AI)-based automated segmentation tool as a pre-processing step for calculating whole-body effective doses to determine the influence of variability in volumetric whole-organ segmentations on dosimetry.

**Procedures:**

PET/CT data of six patients undergoing imaging with ^89^Zr-labelled pembrolizumab were included. Manual organ segmentations were performed, using in-house developed software, and biodistribution information was obtained. Based on the activity biodistribution information, residence times were calculated. The residence times served as input for OLINDA/EXM version 1.0 (Vanderbilt University, 2003) to calculate the whole-body effective dose (mSv/MBq).

Subsequently, organ segmentations were performed using RECOMIA, a cloud-based AI platform for nuclear medicine and radiology research. The workflow for calculating residence times and whole-body effective doses, as described above, was repeated.

**Results:**

Data were acquired on days 2, 4, and 7 post-injection, resulting in 18 scans. Overall analysis time per scan was approximately 4 h for manual segmentations compared to ≤ 30 min using AI-based segmentations. Median Jaccard similarity coefficients between manual and AI-based segmentations varied from 0.05 (range 0.00–0.14) for the pancreas to 0.78 (range 0.74–0.82) for the lungs. Whole-body effective doses differed minimally for the six patients with a median difference in received mSv/MBq of 0.52% (range 0.15–1.95%).

**Conclusion:**

This pilot study suggests that whole-body dosimetry calculations can benefit from fast, automated AI-based whole organ segmentations.

**Supplementary Information:**

The online version contains supplementary material available at 10.1007/s11307-022-01775-5.

## Introduction

As novel tracers are under continuous development, e.g., ^89^Zr-labelled immune tracers, it is important to determine the biodistribution for each newly developed targeting agent and obtain reliable radiation dose estimates. This allows optimization of the amount of activity to be administered while keeping the radiation burden within acceptable limits. Furthermore, timely whole-body dosimetry analysis is crucial for estimating absorbed doses to critical organs prior to, e.g., radioimmunotherapy [1].

After acquiring a series of PET/CT scans, manual whole organ segmentation is typically performed of the organs of interest to obtain specific organ activity biodistribution information for whole-body dosimetry. When using the extracted biodistribution information over time, the area under the time-activity curve extrapolated to infinity represents the total number of disintegrations per unit administered activity (i.e., cumulated activity or residence time) [2]. Next, the mean absorbed dose per organ can be determined using OLINDA/EXM (Organ Level INternal Dose Assessment/EXponential Modeling) software which translates the cumulated activity into absorbed dose using dose conversion factors [3].

Manual organ segmentation is however a very time-consuming task. The importance of faster, simplified (semi-)automated whole organ segmentation methods for dosimetry purposes has been emphasized and explored before [4–7]. Makris et al. (2014) explored various image processing methods. Analysis methods based on spatial volume of interest (VOI) transformation were developed and their influence on residence times and absorbed dose estimates was assessed [5]. In another study by the same group, an active contouring method for semi-automated segmentation of the red marrow, often the dose-limiting organ in radio-immunotherapy studies [8], was developed [4]. More recently, fully automated whole organ segmentation methods for facilitating absorbed dose estimation have emerged, and their performance with respect to manual segmentation has been explored [6, 7]. Depending on the manual segmentations and amount of data used as input to train the network, automated whole organ segmentation methods may also vary in accuracy [9].

Therefore, we decided to perform a pilot study testing an online readily available fully automated artificial intelligence (AI)-based whole organ segmentation tool as a pre-processing step for calculating organ and whole-body absorbed doses to ultimately reduce analysis time. We explored the effect of differences in manual segmentations and AI-based segmentations on the calculated whole-body effective dose estimates.

## Materials and Methods

^89^Zr immuno-PET/CT data of the first six patients participating in the recently published study [10] and acquired between October 2016 and March 2017 were included in the current analysis. The study was approved by the Medical Ethical Committee of the University Medical Center Groningen and registered at ClinicalTrials.gov (NCT02760225). All patients gave written informed consent. Patients in the current analysis received a single injection of ^89^Zr-labelled pembrolizumab and were scanned on day 2, day 4, and day 7 post-injection (p.i.). All images were acquired using a Biograph mCT (Siemens Healthineers, Knoxville, TN, USA) PET/CT system in step-and-shoot mode from the patients’ vertex to feet. Data acquisitions on days 2 and 4 p.i. were performed at 5 min per bed position from the vertex to the upper thigh and imaging of the legs at 2 min per bed position. On day 7 p.i., to account for lower count rates, PET imaging was performed at 10 min per bed position from the vertex to the upper thigh followed by 4 min per bed position for the legs. The acquisition parameters of the low-dose CT were as follows: an X-ray tube current of 99 mAs, a tube voltage of 140 kV, and a spiral pitch factor of 1.5. PET data were reconstructed using the clinically preferred multicentre validated ^89^Zr PET reconstruction protocol [11] using an ordinary Poisson ordered-subset-expectation–maximization 3D-iterative algorithm [12] with 3 iterations, 21 subsets, Time-of-Flight application, resolution modelling, and a Gaussian filter of 8 mm. The resulting image matrix size was 256 × 256 with a voxel size of 3.18 × 3.18 × 2.00 mm.

Subsequently, manual organ segmentations were performed, including a selection of critical organs predefined in the OLINDA VOI definition list [3] using in-house developed software, and biodistribution information was obtained. Manual segmentations were performed by the first author JS with approximately 4 years of experience in image segmentations. These manual segmentations were independently reviewed and corrected, if needed, by two nuclear medicine physicians (AHB and WN) with 25 and 15 years of experience in image reading, respectively. Using the extracted biodistribution information over time, the area under the time-activity curve extrapolated to infinity was calculated, and the total number of disintegrations per unit administered activity (i.e., cumulated activity or residence time) was obtained. The obtained residence times served as input for OLINDA/EXM version 1.0 (Vanderbilt University, 2003) [3] to calculate the effective dose per organ and the whole-body effective dose (mSv/MBq), taking into account tissue weight factors described in the International Commission on Radiological Protection (ICRP) publication 103 [13].

Next, organ segmentations were also performed using RECOMIA (available at: https://www.recomia.org/), a cloud-based AI platform for nuclear medicine and radiology research [14]. This AI tool is based on two convolutional neural networks (loosely inspired by the U-Net) trained on 339 manually segmented CT scans by experienced radiologists [14]; more details can also be found on the RECOMIA website [10]. To test the raw capabilities of the AI tool, segmentations were not reviewed and corrected before further analyses. Moreover, most organs, such as liver, kidneys, spleen, lungs, and brain, are easily visualized on a CT scan and therefore easy to delineate, but this process is mainly time-consuming. Therefore, the AI method is primarily considered to reduce analysis time and final segmentations may need to be supervised and corrected. The subsequent workflow for calculating residence times and whole-body effective doses, as described above, was repeated.

As the available AI-tool offered only a limited amount of organ segmentations per the OLINDA VOI definition list, six organs (brain, kidney, liver, lung, pancreas, and spleen) were included for further analysis in this pilot study.

Volumetric differences per organ between segmentation methods were assessed using a non-parametric Wilcoxon signed-rank test. In addition, Jaccard similarity coefficients were calculated per organ to quantify the extent of organ segmentation similarity between the two methods. A *P*-value of less than 0.05 was considered significant. Using the segmentations as a pre-processing step, the percentage difference between estimated absorbed dose obtained using manual versus AI-based segmentation was compared.

## Results

All six patients included in this analysis had metastatic cancer. There were three men, and three women, 55–72 years of age (median 65 years), with a weight of 46–86 kg (median 68 kg). Patients received an intravenous injection of median 36 MBq (range 35–38 MBq) ^89^Zr-labelled. MAb protein dose for the first two patients was 10 mg (to establish the optimal protein dose for imaging), and the remaining four received 5 mg mAb protein dose. The difference in administered protein doses did not influence organ residence times.

**Fig. 1:**
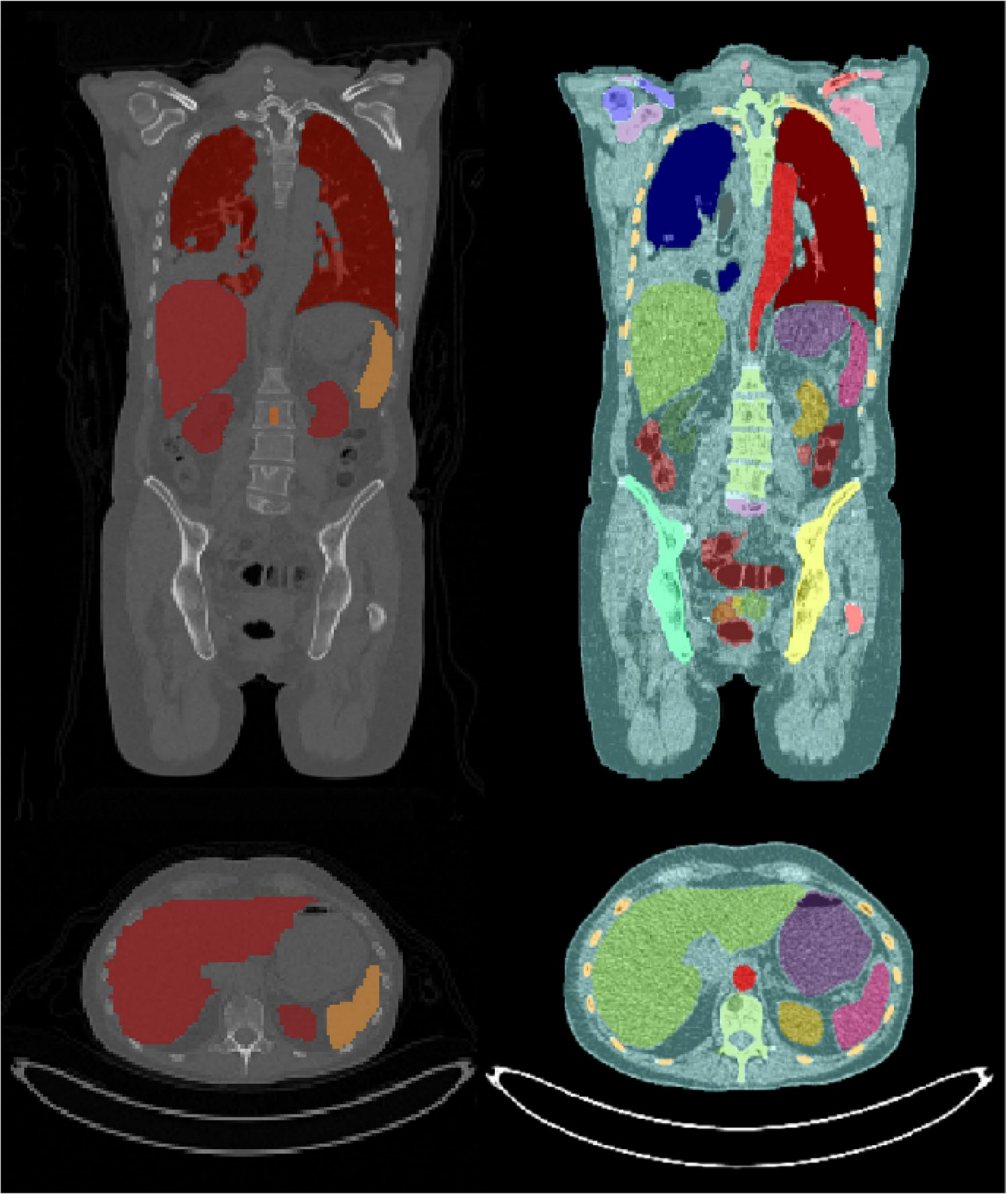
Low-dose CT example patient images in coronal view (upper row) and axial view (lower row) including whole organ segmentations performed manually (left column) and using the AI-based tool (right column)

**Fig. 2:**
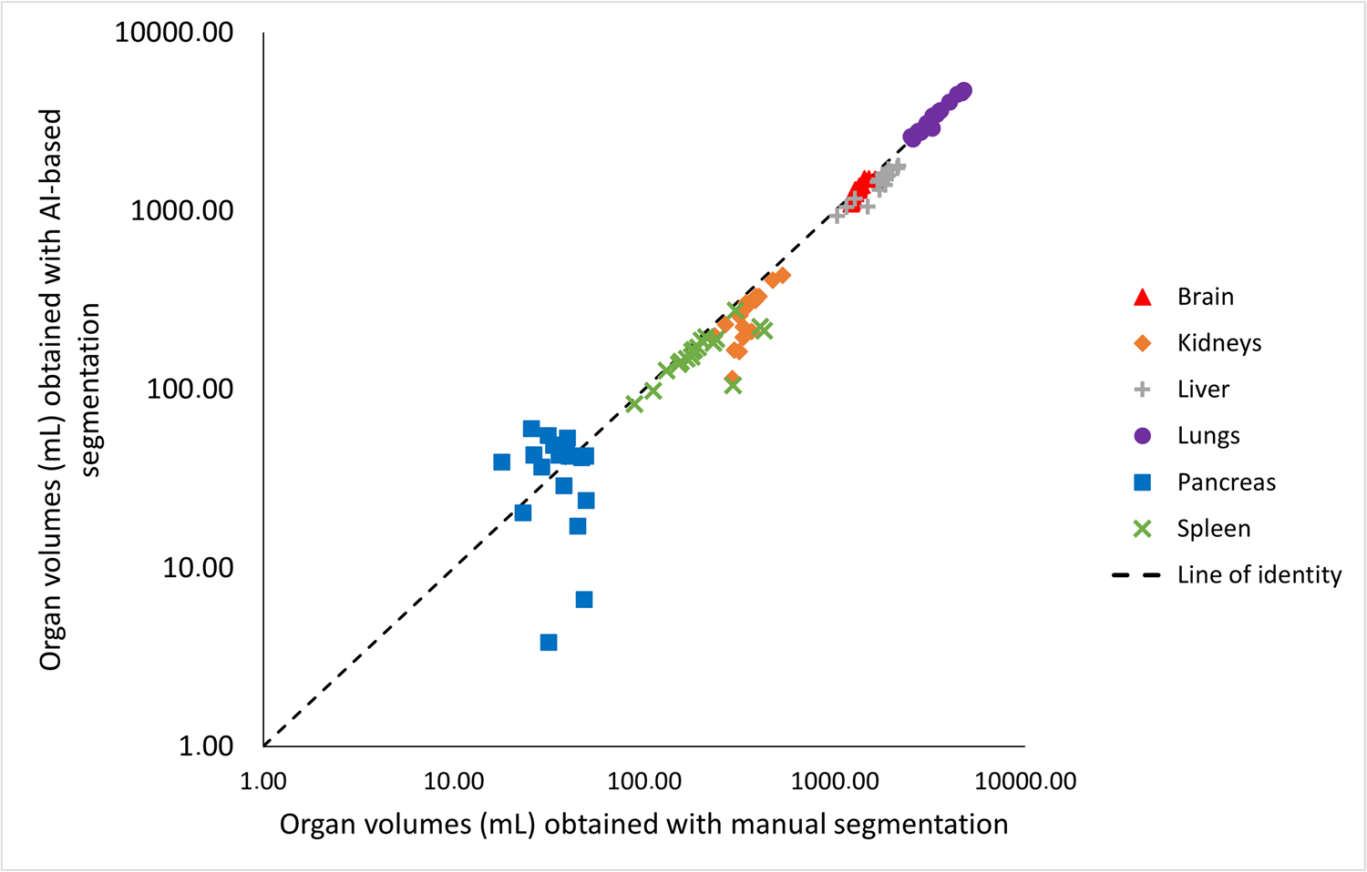
Scatter plot of segmented whole organ volumes obtained using manual segmentation (*x*-axis) and AI-based segmentation (*y*-axis). Axes are in logarithmic scale. Organ volumes obtained using manual and AI-based segmentation were significantly different for the kidneys (*P* = 0.005), the liver (*P* < 0.001), and the spleen (*P* < 0.001)

Overall analysis time per scan incorporating the manual segmentation method was approximately 4 h compared to ≤ 30 min when AI-based segmentation was used. Example patient images, including whole organ segmentations, are shown in Fig. 1. A comparison of organ volumes obtained using the two different segmentations methods is illustrated with scatter plots in Fig. 2. The segmented volumes using manual and AI-based segmentation in the kidneys (*P* = 0.005), in the liver (*P* < 0.001), and in the spleen (*P* < 0.001) were significantly different. Median Jaccard similarity coefficients between manual organ VOI segmentations and AI-based segmentations varied from 0.05 (range 0.00–0.14) for the pancreas to 0.78 (range 0.74–0.82) for the lung. A complete overview of Jaccard similarity coefficients is provided in Table 1.

**Table 1:** Calculated Jaccard similarity coefficients of whole organ segmentations performed manually versus AI-based

	Patient 1			Patient 2			Patient 3			Patient 4			Patient 5			Patient 6		
*Scan day*	1	2	3	1	2	3	1	2	3	1	2	3	1	2	3	1	2	3
Brain	0.53	0.54	0.55	0.53	0.50	0.52	0.53	0.53	0.51	0.56	0.56	0.56	0.43	0.42	0.44	0.48	0.49	0.48
Kidney	0.27	0.13	0.34	0.44	0.38	0.44	0.38	0.36	0.20	0.37	0.36	0.34	0.30	0.31	0.29	0.24	0.16	0.19
Liver	0.67	0.66	0.74	0.72	0.75	0.74	0.74	0.73	0.55	0.73	0.76	0.75	0.71	0.72	0.74	0.70	0.73	0.63
Lung	0.78	0.78	0.80	0.82	0.79	0.82	0.77	0.74	0.74	0.81	0.79	0.78	0.76	0.78	0.77	0.78	0.76	0.77
Pancreas	0.14	0.09	0.14	0.00	0.05	0.00	0.06	0.04	0.06	0.10	0.11	0.10	0.00	0.02	0.00	0.03	0.05	0.01
Spleen	0.52	0.49	0.52	0.53	0.60	0.51	0.46	0.48	0.41	0.44	0.51	0.55	0.48	0.46	0.49	0.30	0.28	0.15

**Fig. 3:**
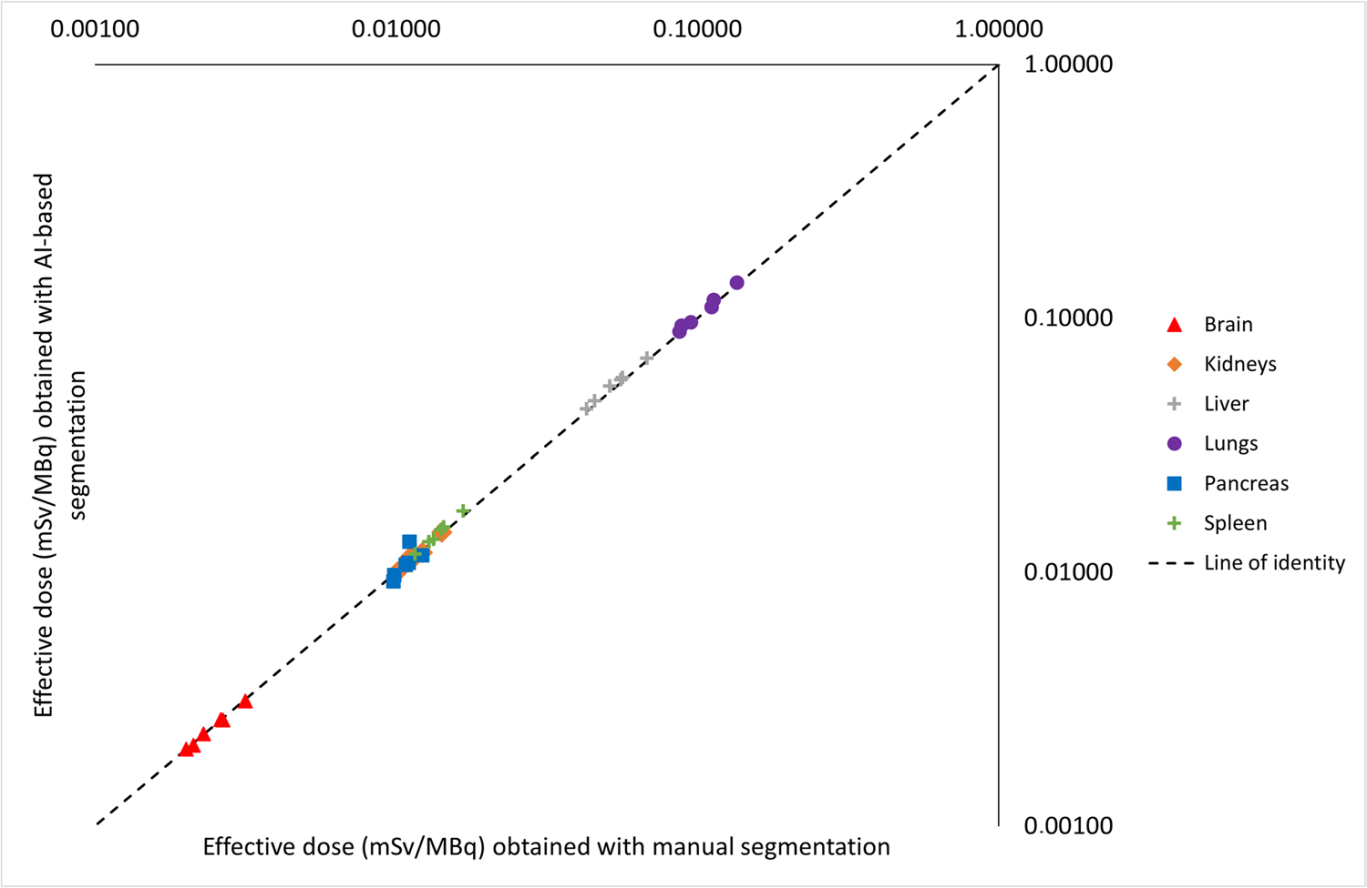
Scatter plot of calculated effective dose per organ obtained using manual whole organ segmentation (*x*-axis) and AI-based whole organ segmentation (*y*-axis). Axes are in logarithmic scale

Calculated whole-body effective doses differed minimally for the six patients with a median difference in received mSv/MBq of 0.52% (range 0.15–1.95%), considering tissue weight factors described in ICRP Publication 103 [13]. A comparison of calculated effective dose per organ obtained using manual and AI-based segmentation is illustrated with a scatter plot in Fig. 3. Whole-body effective doses are shown in Table 2.

**Table 2:** Calculated whole-body effective doses (mSv/MBq) using manual versus AI-based whole organ segmentation as a pre-processing step

Patient #	Manual	AI-based	Difference (%)
1	0.64	0.64	0.15
2	0.51	0.51	0.45
3	0.64	0.64	0.97
4	0.67	0.67	0.59
5	0.52	0.53	1.95
6	0.51	0.52	0.37

## Discussion

It is known that manual segmentations suffer from high interobserver variability. In addition, AI-based segmentations are also subject to variability, e.g., depending on the manual segmentations and amount of data that is used to train the network [9]. Therefore, in the present study, we aimed to explore whether variations in segmented organ volumes would influence estimated effective doses by comparing the dose estimates derived from AI-based based segmentations with those obtained manually.

**Fig. 4:**
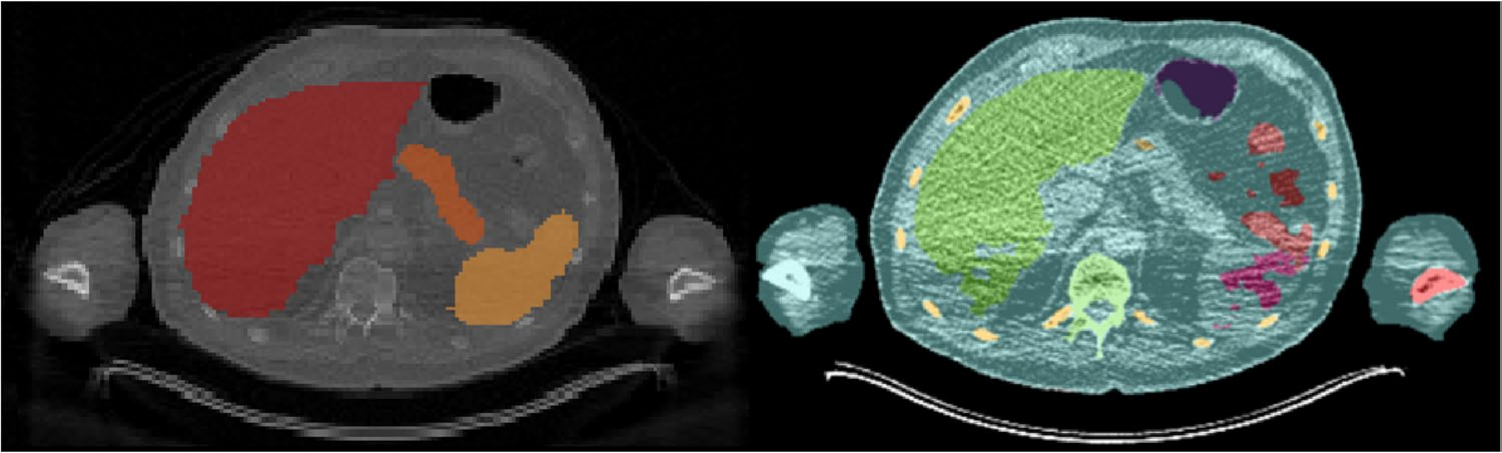
Example axial patient CT images illustrating impaired whole organ segmentations performed using the AI-tool (right) with respect to manual segmentations (left). Poor segmentations by the AI-tool are likely caused by streak artifacts

The manual segmentations performed in this study showed some inaccuracies, for example, inclusion of a small part of the inferior vena cava in the liver segmentation, and exclusion of the border of the kidney, see Fig. 1. Also, the AI-based segmentations showed imperfections, for instance in the kidneys, spleen, and pancreas as can be seen from Jaccard values (Table 1), in Fig. 4, and Supplemental Fig. 1. This pilot study showed however that effective dose estimation is accurate (within 2%) regardless of these variations in manual or AI-based organ segmentation. Since whole-body dosimetry involves the summation of weighted dose values from multiple organs and structures, effective dose calculation is less susceptible to variabilities that arise by the type of segmentation method.

A study by M. Nazari et al. (2021) also used deep learning for automated whole organ segmentation on CT images. They also found a low discrepancy in dose calculations obtained using AI-based and manual segmentation of the kidneys of less than 3% in approximately 40% of the patients and less or equal to 7% in approximately 90% of the patients (*n* = 8) [6]. They mention a specific case that showed 25% deviation in dose estimation obtained using the two segmentation methods. Further exploration of the deviating case showed the discrepancy was due to a larger spill-out effect in the manual segmentation. This illustrates the disadvantage of segmentation by (expert) readers where resulting VOIs are inescapably subject to inter- but also intra-reader variability.

Another comparable study demonstrated a mean organ dose estimation using AI-based versus manual segmentation with less than 5% deviation for the brain, lung, and bone regions. For the eye and parotid gland, the estimated dose differed 7% and 17%, respectively [7]. A multi-atlas averaging was performed to reduce the discrepancy in estimated organ dose with AI-based and manual segmentation [15], resulting in an estimated dose difference of 4% for the eye and 8% for the parotid gland [7]. Another possible approach for future whole organ dose estimation could be to use fast AI-based segmentation as a starting point and, if necessary, adjust the VOIs manually for more accurate whole organ registration.

Despite the small differences in calculated effective doses in the current study, segmented volumes of organs with low contrast to the surrounding tissues, e.g., the pancreas, showed large differences between the manual and AI-based method assessed by the Jaccard similarity coefficient (see Table 1), sometimes even 0.0 indicating no overlap between the differently segmented organ volumes (see Supplemental Fig. 1). The reason for this can be explained two-fold: (1) the contribution of the pancreas to the overall effective dose is small compared to the contribution of all other organs, and (2) there is no clear high uptake of the radiotracer in the pancreas, and thus, larger errors in segmentation will have a minor effect on the effective dose as the contribution from the pancreas, because of low uptake, is small. This behavior was previously confirmed by E. Trägård et al. [14], where they explain that the size of the segmented organ plays a role in the obtained coefficients. The smaller the organ, the more difficult the automated segmentation, i.e., the voxels located at the edge of an organ are difficult to assign to a certain tissue type (e.g., assign to one organ or the other), whereas voxels inside the organ, away from the edge, are easier to classify. Therefore, a higher Jaccard similarity coefficient was expected for large and distinctive organs such as the lungs. However, due to unclear reasons, the AI-based method had trouble capturing the entire organ; an illustrative example is added in Supplemental Fig. 2.

The low-dose CT dataset used in the current study showed streak artifacts in some cases. Example images, including whole organ segmentations, are shown in Fig. 4. These were the cases in which the pancreas was not accurately segmented by the AI-based tool, but also the kidneys, liver, and the spleen showed differences in segmented volume. These volumetric differences did not lead to inaccurate effective dose estimates in this small dataset. However, for smaller or other antibody moieties, accuracy and precision of organ segmentation may be more critical to obtain reliable dose estimates. The developers of the tool confirmed that in the training set of the CNNs, no image artifacts, such as streak artifacts observed in our data, were included. To overcome these issues in future work, a convolutional neural network could be trained to handle these data, including streak artifacts. At this time, we recommend that AI-based segmentations should be supervised by an observer and corrected when needed. Nonetheless, with respect to manual delineation, a considerable amount of time can be saved by supervised AI-based whole organ segmentation.

## Conclusion

With the continuous introduction of novel radiotracers, whole-body effective doses can be assessed rapidly and efficiently through a readily available online AI-based organ segmentation tool (using low-dose CTs) as a dosimetry pre-processing step.

## Supplementary Information

Below is the link to the electronic supplementary material.

Supplementary file1 (DOCX 1846 KB)

## References

[1] Kim JS (2010). Combination radioimmunotherapy approaches and quantification of immuno-PET. Nucl Med Mol Imaging.

[2] Hindorf C, Glatting G, Chiesa C, Lindén O, Flux G (2010). EANM dosimetry committee guidelines for bone marrow and whole-body dosimetry. Eur J Nucl Med Mol Imaging.

[3] Stabin MG, Sparks RB, Crowe E (2005). OLINDA/EXM: the second-generation personal computer software for internal dose assessment in nuclear medicine. J Nucl Med.

[4] Makris NE, Boellaard R, Menke CW, Lammertsma AA, Huisman MC (2016). An automatic delineation method for bone marrow absorbed dose estimation in 89Zr PET/CT studies. EJNMMI Phys.

[5] Makris NE, Van Velden FHP, Huisman MC, Menke CW, Lammertsma AA, Boellaard R (2014). Validation of simplified dosimetry approaches in 89Zr-PET/CT: the use of manual versus semi-automatic delineation methods to estimate organ absorbed doses. Med Phys.

[6] Nazari M, Jiménez-Franco LD, Schroeder M, Kluge A, Bronzel M, Kimiaei S (2021). Automated and robust organ segmentation for 3D-based internal dose calculation. EJNMMI Res.

[7] Schmidt TG, Wang AS, Coradi T, Haas B, Star-Lack J (2016). Accuracy of patient-specific organ dose estimates obtained using an automated image segmentation algorithm. J Med Imaging.

[8] Woliner-van der Weg W, Schoffelen R, Hobbs RF, Gotthardt M, Goldenberg DM, Sharkey RM (2015). Tumor and red bone marrow dosimetry: comparison of methods for prospective treatment planning in pretargeted radioimmunotherapy. EJNMMI Phys.

[9] Renard F, Guedria S, De PN, Vuillerme N (2020). Variability and reproducibility in deep learning for medical image segmentation. Sci Rep [Internet].

[10] The research consortium for medical image analysis (RECOMIA) platform [Internet]. Available from: https://www.recomia.org/. Accessed Nov 2021

[11] Makris NE, Boellaard R, Visser EP, De Jong JR, Vanderlinden B, Wierts R (2014). Multicenter harmonization of 89Zr PET/CT performance. J Nucl Med.

[12] Varrone A, Sjöholm N, Eriksson L, Gulyás B, Halldin C, Farde L (2009). Advancement in PET quantification using 3D-OP-OSEM point spread function reconstruction with the HRRT. Eur J Nucl Med Mol Imaging.

[13] The 2007 Recommendations of the international commission on radiological protection (2007). ICRP publication 103. Ann ICRP.

[14] Trägårdh E, Borrelli P, Kaboteh R, Gillberg T, Ulén J, Enqvist O (2020). RECOMIA—a cloud-based platform for artificial intelligence research in nuclear medicine and radiology. EJNMMI Phys.

[15] Išgum I, Staring M, Rutten A, Prokop M, Viergever MA, Van Ginneken B (2009). Multi-atlas-based segmentation with local decision fusion-application to cardiac and aortic segmentation in CT scans. IEEE Trans Med Imaging.

