# Supplementary material for: Manual Versus Artificial Intelligence-Based Segmentations as a Pre-processing Step in Whole-body PET Dosimetry Calculations
Source: Mol Imaging Biol. 2022 Oct 4;25(2):435–41. doi: 10.1007/s11307-022-01775-5 (PMC10006025; doi:10.1007/s11307-022-01775-5)
Supplement: Supplementary file 1 — Supplementary file1 (DOCX 1846 KB) [file 11307_2022_1775_MOESM1_ESM.docx]

**SUPPLEMENTAL DATA**


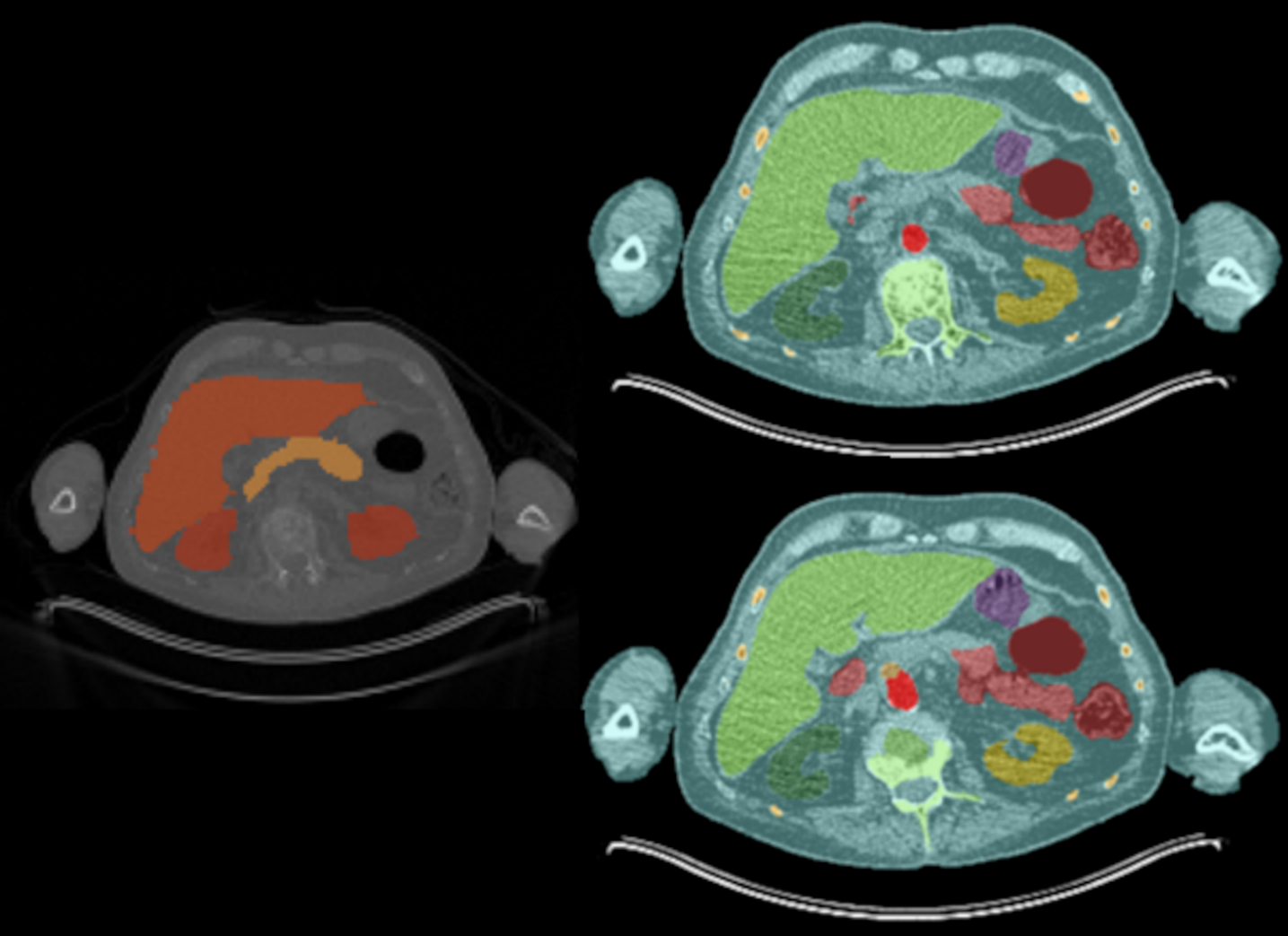


**Supplemental Fig. 1** Low dose CT example patient images in axial view including whole organ segmentations performed manually (left) and using the AI-based tool (right) illustrating how the AI-based tool is not always successful in delineating the pancreas (in light orange).


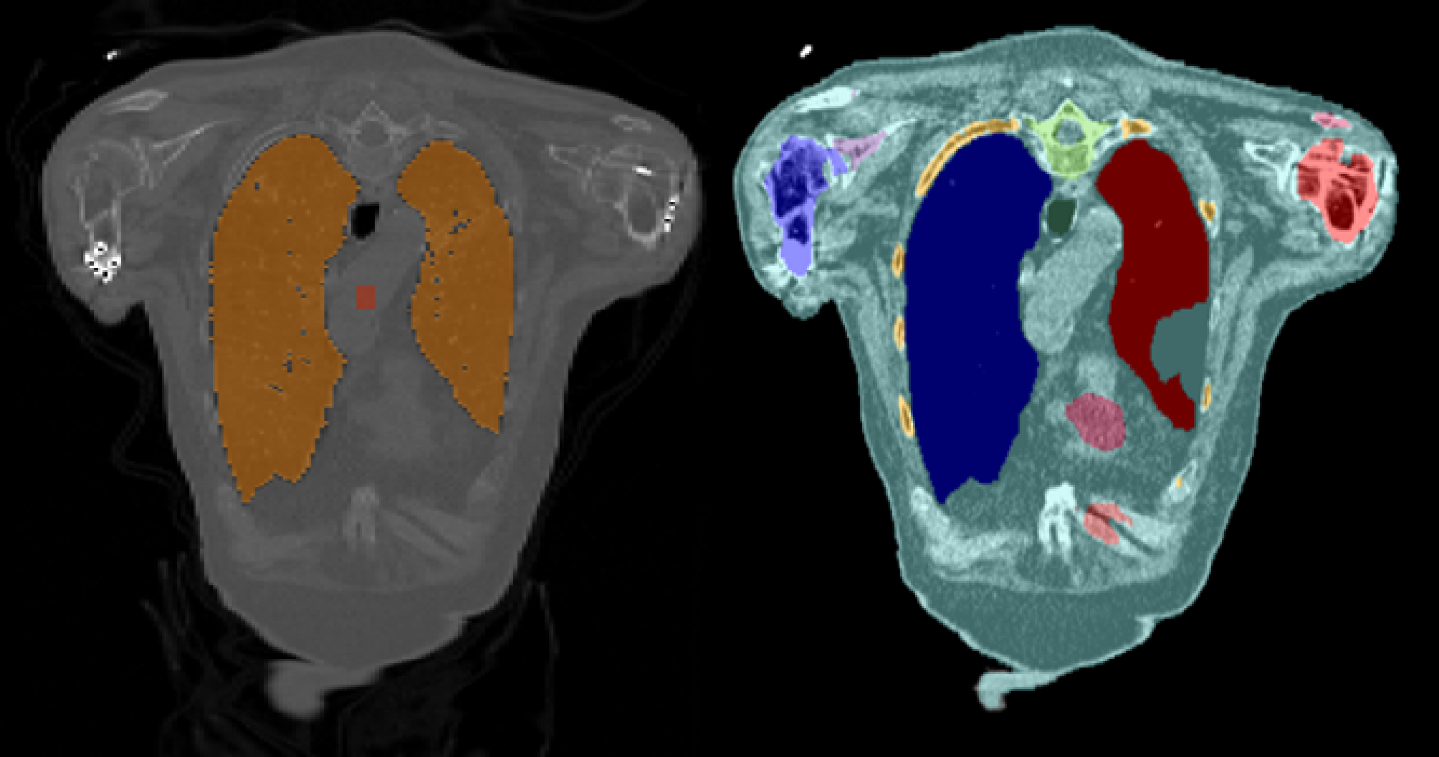


**Supplemental Fig. 2** Low dose CT example patient images in coronal view including whole organ segmentations performed manually (left) and using the AI-based tool (right) illustrating how the AI-based tool is not always successful in delineating the entire lung.
